# An Examination of Dental Health Among Metropolitan and Appalachian Adolescents in Ohio

**Published:** 2019-12

**Authors:** Kyle Bader, Megan E. Roberts, Brittney Keller-Hamilton

**Affiliations:** 1 College of Dentistry, The Ohio State University, Columbus, OH; 2 Division of Health Behavior and Health Promotion, College of Public Health, The Ohio State University, Columbus, OH; 3 Division of Epidemiology, College of Public Health, The Ohio State University, Columbus, OH

**Keywords:** Appalachia, Dental health, Diet, Tobacco

## Abstract

**Background:**

Poor dental health is a common chronic condition among youth. Appalachian versus metropolitan residence, socioeconomic status, and health behaviors contribute to poor dental health. Limited research has directly compared dental health and risk factors for poor dental health among Appalachian and metropolitan youth. We examined the association between dental health and residence among adolescent boys and explored socioeconomic and behavioral factors that may contribute to differences in dental health.

**Methods:**

Adolescent males from metropolitan and rural Appalachian Ohio (n = 1220, age 11-16 years) reported their diet and tobacco use. Parents or guardians reported when boys had last visited the dentist and rated their dental health (excellent/very good/good versus fair/poor). Unadjusted logistic regression modeled the association between fair/poor dental health and residence (metropolitan versus Appalachian). Adjusted analyses controlled for race, household income, dental visits, diet, and tobacco use.

**Results:**

Appalachian (versus metropolitan) boys were more likely to have used tobacco in the past 30 days and consumed fewer fruit and vegetables, more added sugar, and more sugary beverages. The relation between dental health and Appalachian versus metropolitan residence did not reach statistical significance, and adjusting for behavioral factors did little to change the observed association.

**Conclusion:**

Our findings suggest that some of the urban/rural disparities in dental health observed in other studies may be related to behavioral factors like tobacco use and diet, but much remains unexplained. We provide support for behavioral interventions to address these issues in the Appalachian community.

## INTRODUCTION

Good dental health, or the absence of dental decay and excessive tooth loss, is essential to good general health.^1^ Although good dental health should be considered a priority in order to promote good general health, many children and adolescents are not meeting this goal. From 2011 through 2014, for example, 18.6% of youth had untreated dental caries,^1^ which is one of the most common childhood chronic conditions.^2^ A few years earlier, from 2005 through 2008, approximately 1 in 5 children and 1 in 7 adolescents had at least 1 untreated decayed tooth.^2^

A variety of factors affect overall dental health, including socioeconomic status, health behaviors, and geography. Regarding socioeconomic status, children aged 5 to 9 years from low-income backgrounds are more than twice as likely to have dental caries than children from higher income backgrounds.^3^ Dental insurance quality and type may affect dental health. In Ohio, Medicaid covers 40% of all children,^4^ and historically many dental professionals did not accept Medicaid.^5^ However, expansions of Medicaid coverage have more recently been associated with better dental health and improved access to care among low-income and racial and ethnic minority populations.^6–8^ Additionally, observed differences in dental care utilization between children with private versus public insurance are attenuated when confounding factors like overall health status and poverty level are controlled.^9^ However, parents’ satisfaction with their child’s dental care is low when their child is enrolled in Medicaid, and low satisfaction is associated with not having a regular source of dental care.^10^ Travel distance and lack of public transportation may also contribute to poor dental health within low-income rural and urban communities.^11,12^ Low health literacy might contribute to differences in dental health outcomes according to socioeconomic status, although results are contradictory and associations are generally weak.^13^

Health behaviors, like diet, tobacco use, and oral hygiene are also related to dental health among children and adolescents. Diet plays a major role in dental health, especially for tooth decay.^14^ Moreover, the World Health Organization reported a positive association between increased consumption of free sugars, monosaccharides, and disaccharides that are added to food and sugars naturally present in honey, syrups, and juices and the increased prevalence of dental caries across all ages.^15^ Tobacco use also leads to dental health issues including periodontal disease^16^ and dental caries among adolescents.^17^ Inadequate oral hygiene is another risk factor for poor dental health, especially among adolescents.^18^

Geography is related to dental health, as Appalachian residents are less likely to utilize dental care and have worse dental health than their metropolitan counterparts on average.^19^ One cause of reduced dental care utilization in Appalachian areas may be related to fear of visiting the dentist. A study conducted in West Virginia, an entirely Appalachian state with known dental health disparities, reported a high prevalence (47.1%) of dental fear. Importantly, dental fear was associated with having delayed dental care appointments.^20^ Dental health might also be less emphasized in Appalachian compared to metropolitan communities; in West Virginia, the need for good dental health was ranked as the lowest priority out of other health issues such as obesity, cancer, and alcohol and drug use.^21^ Overall, poor diet, which includes sugary beverages, “low” fruit and vegetable intake, and added sugar, is more prevalent in Appalachian communities compared to metropolitan communities.^22^ Also, the prevalence of tobacco use is greater in Appalachian adolescent populations compared to metropolitan adolescent populations.^23^

Although research has examined the overall health differences between Appalachian and metropolitan youth, few studies have directly compared these populations with respect to dental health status. Moreover, in spite of the interplay between geography, socioeconomic status, and health behaviors, examination of these factors in the same study has not been conducted to our knowledge. Understanding how these factors contribute to dental health among male youth has implications for public health in Ohio, which contains both Appalachian and metropolitan populations. The current study had the following objectives: (1) estimate the association between male adolescent dental health and Appalachian versus metropolitan residence and (2) examine how other demographic, socioeconomic, and behavioral risk factors affect the association between geography and adolescent dental health.

## METHODS

### Participants

Data came from the Buckeye Teen Health Study (BTHS), a sample of 1220 adolescent boys aged 11 to 16 years who resided in Franklin County, Ohio (N = 708), or 1 of 9 Appalachian Ohio counties (N = 512). Franklin County, which includes the city of Columbus, is designated metropolitan by the Office of Management and Budget. Appalachian Ohio counties were designated Appalachian by the Appalachian Regional Commission and included Brown, Clermont, Guernsey, Lawrence, Morgan, Muskingum, Noble, Scioto, and Washington. Only males were included in the study because an aim of the parent study was to measure predictors of smokeless tobacco use. Participants were recruited through probability address-based sampling (N = 991) and nonprobability convenience sampling (N = 229), including advertising at community events, snowball sampling, and advertising in various media outlets. Additional information about sampling and recruitment procedures are provided elsewhere.^24^

### Setting and Design

The BTHS is a longitudinal cohort study; only baseline data are reported here. Thus, the current study is cross-sectional. At baseline, trained interviewers, residing in the same region as the participant, obtained informed permission and assent from parents/guardians and adolescent participants, respectively. Non-sensitive items, including age, race, and diet, were interviewer-administered. Sensitive items, including tobacco use, were administered using audio computer-assisted self-interviewing (ACASI). When permitted, participants were separated from their parents/guardians when completing the ACASI portion of the survey. Parents/guardians completed a self-administered questionnaire to provide information about participants’ dental health, visits to the dentist, and household income. The Institutional Review Board at The Ohio State University approved all study procedures.

### Measures

#### Outcome Variable

The primary outcome variable of this study was parent/guardian-reported dental health of the male youth participants, which was assessed with the question,^25^ “How would you describe the condition of your son’s teeth: excellent, very good, good, fair, or poor?” Responses were dichotomized as excellent/very good/good versus fair/poor.

#### Predictor Variable

The primary predictor variable was the social environment variable of living in metropolitan versus Appalachian Ohio. This variable was assessed upon sampling.

#### Risk Factors

Additional risk factors included dental visits, diet, tobacco use, and demographic variables. Parents/guardians reported dental visits with the item, “About how long has it been since your son visited a dentist? Include all types of dentists, such as orthodontists, oral surgeons, and all other dental specialists, as well as dental hygienists.” Responses were dichotomized as less than a year and more than a year since last visiting the dentist. Participants’ diets were assessed using the Block Kids 2004 Food Frequency Questionnaire (FFQ) from NutritionQuest.^26^ Briefly, the FFQ asked participants to report how many days they consumed different foods and beverages in the past week and the typical serving size of each food or beverage. From the FFQ results, total cups of fruits and vegetables, teaspoons of added sugars, and average grams of sugary beverages were used. Participants’ tobacco use was assessed separately for cigarettes, smokeless tobacco, electronic cigarettes, pipes, cigars, hookah, bidis, and kreteks, and participants who were tobacco users reported the last time they used each product. Product-specific results were combined to use of any tobacco product in the past 30 days (yes versus no). Demographic variables included age, race/ethnicity (white non-Hispanic, black non-Hispanic, and other), and household income (<$50000 versus $50000 or more).

### Statistical Analysis

All analyses were survey-weighted to reflect the sampling design; details about weighting procedures are provided elsewhere.^24^ Missing values of tobacco use or race/ethnicity were imputed using hot deck single imputation (<9% missing). Stratification variables for the hot deck imputation included age at enrollment (11-12, 13-14, and 15-16 years), residence, and household adult tobacco use. Participants who were missing parent-reported dental data were excluded from the analysis (N = 22). Most of the missing data were due to parents not enrolling in the study, and one parent responded “Don’t Know” for both perceived dental health and dental visits.

Our analyses first described the distributions of age, race/ethnicity, dental visits, tobacco use, and diet overall and by metropolitan versus Appalachian residence. Second, we used Rao-Scott chi-square tests and linear regression models to estimate the associations between the predictor variable (ie, residence) and risk factors (ie, dental visits, diet, tobacco use, age, race/ethnicity, and income). Third, we used logistic regression to model the unadjusted association between parent/guardian-rated dental health and metropolitan versus Appalachian residence. Finally, we sequentially added risk factors to the model to estimate adjusted effects. The first model controlled for demographics and tobacco use. The next 3 models added 1 dietary variable at a time to avoid multicol-linearity (ie, cups of fruits and vegetables, teaspoons of added sugars, and grams of sugary beverages were added separately to the first adjusted model). Finally, we fit the fifth model which included all risk factors except for sugary beverage intake due to its collinearity with added sugar intake. Only risk factors that were substantively or statistically associated with residence in the bivariable analyses were included in the adjusted models.

An alpha level of 0.05 was used for statistical significance. SAS version 9.4 (SAS Institute Inc., Cary, NC) was used to analyze the data.

## RESULTS

### Participant Characteristics

Overall, after applying survey weights, male youth were aged 14 years on average, 71.2% were white non-Hispanic, 33.8% had a total household income less than $50000, and 73.6% were from metropolitan Franklin County, Ohio. An estimated 92.8% of boys overall had dental health that was rated by parents as excellent/very good/good, 90.7% had visited the dentist in the past year, and 4.9% had used a tobacco product in the past 30 days. On average, boys consumed 15.6 ± 0.38 teaspoons of added sugar, 2.98 ± 0.08 cups of fruits and vegetables, and 310.8 ± 11.4 grams of sugary beverages per day.

### Risk Factors

Compared to metropolitan boys, Appalachian boys were more likely to be white non-Hispanic (Table 1; *P* <0.001), have used tobacco in the past 30 days (*P* = 0.006), and have a household income less than $50000 (*P* <0.001). Boys living in Appalachia also consumed fewer fruits and vegetables (*P* = 0.001), more added sugar (*P* <0.001), and more sugary beverages than their metropolitan counterparts (*P* <0.001).

### Metropolitan Versus Appalachian Residence and Dental Health

The association between parent/guardian-rated dental health and residence was not statistically significant, although the trend was for boys residing in metropolitan areas to be more likely to have excellent/very good/good dental health than those in Appalachia (Table 1; *P* = 0.07). Results of unadjusted and adjusted logistic regression models that estimated the odds of fair/poor dental health are presented in Table 2. In the unadjusted logistic regression model, boys in Appalachian Ohio had somewhat higher odds of fair/poor dental health than boys in metropolitan Ohio, but the odds ratio did not reach statistical significance (OR = 1.61; 95% CI: 0.97, 2.67); they also did not reach statistical significance after accounting for race/ethnicity, household income, tobacco use, and dental visits, or when further controlling for diet variables individually. Age was not controlled for in any models because it was not substantively or statistically associated with parent-reported dental health or metropolitan versus Appalachian residence.

## DISCUSSION

This study found an association between rural residence and several risk factors for poor oral health. Regarding diet, Appalachian adolescents consumed fewer total fruit and vegetables, but more added sugar and sugary beverages. We did not find support for an association between having visited the dentist in the past year and residence in the current study, with the prevalence of having visited the dentist in the past year being around 90% in both areas. Yet the prevalence of past 30-day tobacco use was higher among adolescents in Appalachia. These findings suggest that some behavioral and dental health risk factors might contribute to the dental health differences between urban and rural populations that have been found in other studies. However, when directly comparing the dental health of Appalachian and metropolitan adolescents in Ohio, we only found that Appalachian adolescents had a marginally higher proportion of fair or poor dental health than metropolitan adolescents. It is possible that significant dental health differences will emerge between these populations as they age, but that is a question for future research.

Our findings that multiple risk factors for marginally higher odds of fair or poor dental health were associated with residence agree with existing literature on rural disparities. In our study, household income was lower among the Appalachian compared to metropolitan boys. Other work in Appalachia has reported that more than one-fourth of households had an income of less than $10000, and more than half earned $30000 or less.^27^ Regarding tobacco use, Appalachian males in our study were more likely to use any tobacco product in the past 30 days. In 2017, past 30-day tobacco use among adolescents aged 12 to 17 years in nonmetropolitan areas was 7.8%, while it was 3.8% in large metropolitan areas and 5.6% in small metropolitan areas.^23^ Moreover, rural adolescents were more likely to become daily smokers than urban and suburban adolescents.^28^ Additionally, Appalachian populations in Ohio are more likely to consume a poor diet compared to African American and white urban populations in Ohio.^22^

Because access to dental care is a major barrier to proper dental health, we examined whether having visited a dentist in the past year was associated with metropolitan versus Appalachian residence. Our findings showed that there was no difference in frequency of dental visits by residence. Interestingly, previous research has found that rural residents were less likely to visit the dentist in the past year.^29^ An explanation for this difference could be due to all participants living in Ohio and not fully being representative of all rural areas in the country. Because Appalachian residents in Ohio could nonetheless live fairly close to more populous Ohio cities with pediatric dentists, distance might not be a major barrier to receiving care for our study participants. Another reason could be that most of our Appalachian counties included midsized cities that had at least a few practicing pediatric dentists. An additional possible explanation could be Ohio’s Medicaid expansion in 2013. From preexpansion in 2012 to postexpansion in 2015, the prevalence of uninsured Ohio children decreased by half (4.6% to 2.2%).^30^ While coverage for children is normally high, the expansion could have made visiting the dentist more feasible for some of our participants.

### Strengths and Limitations

A key strength of the study was the large sample that was representative of metropolitan and Appalachian adolescent boys in our study counties, which allowed us to directly compare dental health between groups. The sample was survey-weighted and adjusted during the analysis to represent the target population. Another strength was that we were able to examine many variables that appear to explain some of the association between dental health and metropolitan versus Appalachian residence.

A major limitation of this study was that the participants’ dental health status was obtained from the parent/guardian-reported survey; a more valid diagnosis of dental health would come from dental professionals or use of a comprehensive, validated self-administered scale (eg, the short-form oral health impact profile^31^). In fact, it is possible that using parent/guardian-reported dental health contributed to our marginally significant findings. For example, given the perceived low susceptibility to poor dental health among Appalachian adults,^11^ it is possible that Appalachian parents might have been less likely to rate their child’s dental health as poor than metropolitan parents in cases where the youth had the same dental health. As dental health was not the focus of the parent study, we also lacked additional oral health variables.

Another limitation was that we used cross-sectional data and were therefore unable to determine the temporality of our outcome and predictor variables. The FFQ, for example, inquired about foods and beverages consumed in the past week. Therefore, we had to assume that the adolescent’s diet over the past week was representative of his diet when his level of dental heath was being established. Finally, data were obtained from a parent study which focused on smokeless tobacco use and thus was restricted to boys. Therefore, we were not able to identify possible differences in dental health and behavioral factors between genders. Further, there were only a few dental health questions asked and we could not gather a more comprehensive analysis of dental health and dental risk factors in relation to metropolitan versus Appalachian residence, such as number of times participants brush and floss per day.

## PUBLIC HEALTH IMPLICATIONS

Disparities in risk factors for poor dental health between metropolitan and Appalachian Ohio adolescents are a public health problem. The Ohio Department of Health determined that 7 of the 11 counties with the poorest dental health in the state were Appalachian.^32^ Furthermore, the prevalence of untreated cavities among Appalachian adolescents (27%) is greater than in the rest of Ohio (16%) and 69% higher than in urban or suburban areas.^32^ Our results identify some risk factors that could potentially be addressed to improve dental health among Appalachian adolescents, including tobacco prevention and nutrition interventions.

Regarding tobacco prevention programs, many localities in Ohio, including Columbus in Franklin County, have adopted a Tobacco 21 law over the past few years, which prohibits all sales of tobacco products and paraphernalia to those under 21 years of age.^33^ Recently, the state of Ohio also adopted Tobacco 21 policy. Tobacco 21 laws have been shown, in some cases, to reduce the prevalence of tobacco purchases among adolescents.^34^ However, the effect of Tobacco 21 in Ohio has not yet been evaluated.

In order to target Appalachian adolescent populations, community and policy interventions in school meals could help improve the nutritional intake among students. The National School Lunch Program and the Ohio School Breakfast Program allow students to have free or reduced-price school meals based on multiple factors surrounding the household income.^35^ Therefore, improving the nutritional standards of school lunches could help improve the dietary choices made by students, especially those who qualify for the program.^36^ Considering Appalachian households have lower incomes, these families might benefit most from these programs. Dental practices also provide a natural setting to counsel both metropolitan and Appalachian Ohio adolescents about the dangers of tobacco use and poor nutrition to dental health.

The Ohio Department of Health has dental health care programs to help residents who struggle to access the proper care. Safety net dental programs include public dental clinics, dental programs in schools, and mobile programs which provide dental care to Medicaid patients and those who cannot afford a private dentist. Though this improves dental health care accessibility, some Appalachian counties do not have these clinics, and some require the patient to be a resident of the county to receive care.^37^ Increased funding directed toward opening more safety net dental clinics in counties that do not have them would decrease travel obstacles as well as provide an opportunity for dental health professionals to counsel patients of all ages on good dental health practices.

## Figures and Tables

**Table 1.** Distribution of Parent-Reported Dental Health and Risk Factors by Metropolitan and Appalachian Residence, Ohio, 2015-2016^a^

	Metropolitan (n = 708)	Appalachian (n = 512)

**Parent-reported dental health (%)**		
Excellent, very good, or good	93.7	90.3
Fair or poor	6.3	9.7

**Dental visits (%)**		
Over 1 year ago	8.7	11.0
Within past year	91.3	89.0

**Dietary variables (mean ± SEM)**		
Total fruit and vegetables*	3.1 ± 0.1	2.6 ± 0.1
Added sugar*	14.6 ± 0.5	18.3 ± 0.6
Sugary beverage*	276.8 ± 13.6	404.6 ± 19.8

**Past 30-day tobacco use (%)** *		
Not used in 30 days	96.2	92.0
Used in 30 days	3.8	8.0

**Age (%)**		
11-13 years	51.3	50.0
14-16 years	48.7	50.0

**Race/Ethnicity (%)** *		
Non-Hispanic White	64.0	91.4
Non-Hispanic Black	22.0	1.6
Other	14.0	7.0

**Household Income (%)** *		
At or above $50000	70.0	55.3
Below $50000	30.0	44.7

Abbreviations: SEM = standard error of the mean

* *P* < 0.01

a Means and proportions are survey-weighted; unweighted subject counts are reported.

**Table 2.** Odds of Fair/Poor Dental Health Among Adolescent Boys in Metropolitan and Appalachian Ohio, 2015-2016^a^

	Model 1^b^ OR (95% CI)	Model 2 ^b^ OR (95% CI)	Model 3 ^b^ OR (95% CI)	Model 4^b^ OR (95% CI)	Model 5 ^b^ OR (95% CI)	Model 6^b^ OR (95% CI)

**Residence**						
Metropolitan	1.00	1.00	1.00	1.00	1.00	1.00
Appalachian	1.61 (0.97-2.67)	1.79 (0.96-3.33)	1.73 (0.93-3.21)	1.74 (0.95-3.19)	1.70 (0.92-3.13)	1.58 (0.86-2.90)

**Race/Ethnicity**						
White		1.00	1.00	1.00	1.00	1.00
Black		2.02 (0.89-4.56)	2.14 (0.95-4.78)	2.09 (0.93-4.70)	2.08 (0.93-4.67)	2.12 (0.95-4.74)
Other		0.79 (0.26-2.41)	0.83 (0.27-2.50)	0.79 (0.26-2.41)	0.79 (0.26-2.39)	0.85 (0.28-2.61)

**Household income**						
<$50000		1.00	1.00	1.00	1.00	1.00
≥$50000		0.66 (0.35-1.23)	0.65 (0.35-1.22)	0.66 (0.35-1.23)	0.67 (0.36-1.25)	0.69 (0.37-1.28)

**Past 30-day tobacco use**						
No		1.00	1.00	1.00	1.00	1.00
Yes		1.13 (0.40-3.23)	1.05 (0.37-3.01)	1.08 (0.37-3.10)	1.07 (0.37-3.09)	1.00 (0.35-2.88)

**Dental visits**						
Within year		1.00	1.00	1.00	1.00	1.00
>1 year ago		1.95 (0.85-4.44)	1.92 (0.83-4.45)	1.90 (0.83-4.35)	1.89 (0.82-4.34)	1.93 (0.83-4.49)

**Fruit/Vegetable intake (per cup)**			0.91 (0.82-1.02)			0.89 (0.79-1.00)
**Sugary beverage intake (per gram)**				1.00 (1.00-1.01)		
**Added sugar intake (per teaspoon)**					1.01 (0.99-1.03)	1.02 (1.00-1.04)

Abbreviations: OR=odds ratio; CI=confidence interval

a Logistic regression models were survey-weighted to represent the sampling design.

b Model 1 is the unadjusted model that includes residence only. Model 2 includes residence and controls for race/ethnicity, household income, dental visits in the last year, and any tobacco use. Model 3 includes the variables in Model 2 plus fruit and vegetable intake. Model 4 includes the variables in Model 2 plus total sugary beverage intake. Model 5 includes the variables in Model 2 plus added sugar intake. Model 6 includes the variables in Model 2 plus fruit and vegetable intake and added sugar intake.
